# Translational Success and Pharmacoeconomic Lessons of Pandemic-Driven Drug Repurposing

**DOI:** 10.7759/cureus.85033

**Published:** 2025-05-29

**Authors:** Kelechi W Elechi, Adekunle F Adeoye, Aliyu O Olaniyi, Olukunle O Akanbi, Isaiah Olumeko, Chukwuma G Udensi, Toluwanimi J Kolapo, Vincent U Barrah

**Affiliations:** 1 Integrated Biomedical Sciences, University of Texas Health Science Center at San Antonio, San Antonio, USA; 2 Mathematics and Statistics, Georgia State University, Atlanta, USA; 3 Geriatrics, Stepping Hill Hospital, Manchester, GBR; 4 Graduate College of Business Leadership, National Louis University, Florida, USA; 5 Pharmaceutical Health Outcomes and Policy, University of Houston, Houston, USA; 6 Molecular Biosciences, University of Kansas, Lawrence, USA; 7 Biology, Miami University, Oxford, USA; 8 Public Health, Chicago State University, Chicago, USA

**Keywords:** adaptive platform trials, covid-19, dexamethasone, drug repurposing, pharmacoeconomics, remdesivir, translational science

## Abstract

The COVID-19 pandemic spurred an unprecedented wave of drug repurposing as scientists and clinicians raced to find immediate treatment options for a novel disease. This narrative review examines how those crisis-driven repurposing efforts fared. It highlights key successes and failures in translating research into practice and assessing their pharmacoeconomic implications in high-income health systems. It also distills lessons to guide future pandemic preparedness and improve equitable global access to effective treatments. We performed a broad literature search across major databases (2020-2025) to identify studies and reports on repurposed COVID-19 therapies and health economic outcomes. While repurposing accelerated the delivery of treatments, results were mixed: a handful of existing drugs, such as the widely available steroid dexamethasone, that reduced mortality, emerged as life-saving interventions, but many other initially promising drugs ultimately showed limited or no efficacy. Agile translational research frameworks like large adaptive trials proved critical, separating truly effective therapies from many speculative candidates. From a pharmacoeconomic perspective, repurposed therapies yielded cost-effective breakthroughs and costly disappointments. High-income countries invested substantial resources in repurposed drugs. In some cases, this approach provided rapid access to evidence-based care but also led to significant spending on unproven interventions, underscoring the importance of timely evidence generation and prudent resource allocation. Disparities in access to effective therapies between wealthy and low-resource settings highlight a persistent global equity challenge. The collective experience of pandemic drug repurposing provides a pragmatic blueprint for balancing urgency with scientific rigor, economic prudence, and equity. This will ultimately guide how we might better pivot from crisis to cure in future global health emergencies.

## Introduction and background

The COVID-19 pandemic has served as an unprecedented global stress test for translational science. Virtually overnight, researchers and clinicians worldwide were challenged to translate benchside insights into bedside interventions at a scale and speed never before demanded. In the face of an emergent virus and mounting casualties, traditional drug development timelines proved untenable. This crisis environment forced the medical community to think creatively and act decisively, accelerating innovations and collapsing the usual barriers between laboratory discoveries and clinical application. Now, looking back at this period, it is evident that the pandemic not only tested our scientific agility but also showcased the potential of adaptive strategies when confronting a rapidly evolving global health threat.

A central strategy that emerged early in the pandemic was drug repurposing, the search for new therapeutic uses of existing drugs. Drug repurposing (often used interchangeably with drug repositioning) offered a pragmatic shortcut: instead of formulating brand-new therapies from scratch, it focused on leveraging medications already known to be safe in humans [1]. Some experts distinguish the terms, with repositioning sometimes implying a deliberate shift of a drug into a new indication or patient population, but both concepts revolve around the same idea. By redeploying approved or investigational agents for COVID-19, scientists capitalized on accumulated knowledge, hoping to find effective treatments in a fraction of the time normally required. This approach led to a flood of clinical trials testing everything from antivirals developed for other infections to anti-inflammatory drugs used in chronic diseases, reflecting a collective attempt to find any lifesaving benefit hidden in our pharmacological arsenal.

**Figure 1:**
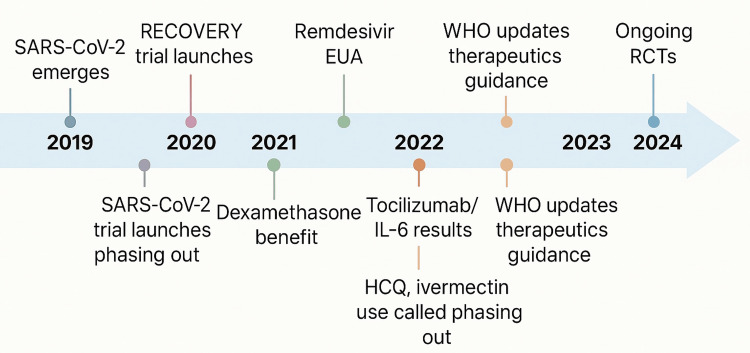
Pandemic timeline of repurposed drug milestones (2019 – 2024) Chronological overview of major COVID‑19 repurposing events: SARS‑CoV‑2 emergence; launch of adaptive trials such as the Randomised Evaluation of COVID‑19 Therapy (RECOVERY) platform in March 2020; Emergency Use Authorization (EUA) of remdesivir in May 2020; publication of the dexamethasone mortality benefit in June 2020; interleukin‑6 (IL‑6) inhibitor results (tocilizumab) in 2021; successive World Health Organization (WHO) therapeutic guideline updates (2021–2022); withdrawal of ineffective agents such as hydroxychloroquine (HCQ) and ivermectin; and ongoing randomised controlled trials (RCTs) through 2024.

Alongside the scientific urgency came a keen awareness of pharmacoeconomics, the cost-effectiveness and value assessment of therapies [2], especially pronounced in high-income countries (HICs). Even well-resourced health systems faced strained budgets and critical allocation decisions as the pandemic surged. The economic dimension of drug repurposing became impossible to ignore: some of the most notable therapeutic successes were inexpensive generics, a fact that carried enormous implications for access and healthcare expenditure. An old corticosteroid (dexamethasone), for example, emerged as one of the first proven life-saving treatments for severe COVID-19 - a triumph not only of translational science but of value-driven care [3,4]. Conversely, several cutting-edge treatments offered only modest benefits at substantially higher costs, underscoring the importance of weighing financial considerations alongside clinical efficacy. These realities highlighted that in HICs, where advanced therapies are available but resources are still finite, robust pharmacoeconomic evaluation is integral to pandemic response planning. Figure 1 charts the rapid sequence of trial launches, regulatory decisions, and guideline updates that shaped COVID‑19 drug repurposing between 2019 and 2024.

In this narrative review, we aim to examine how the COVID-19 crisis propelled drug repurposing from urgent hypothesis to clinical practice and to derive pharmacoeconomic lessons from these efforts. We will outline the translational successes achieved under pandemic pressures and analyze how cost-benefit considerations influenced decision-making in resource-rich healthcare settings. The review is organized to first frame the landscape of pandemic-driven repurposing efforts, then highlight key case examples of repurposed drugs and their outcomes. By clearly delineating these facets, the article sets out to illuminate how a crisis catalyzed innovative cures and what this means for the future of translational science and healthcare economics in the face of future global health emergencies.

## Review

Narrative search approach

We conducted a comprehensive literature search across major databases, including PubMed, medRxiv, and the WHO Global COVID-19 Database, to identify relevant publications from December 2019 through March 2025. This strategy encompassed peer-reviewed articles and select high-impact preprints to capture the most up-to-date evidence on pandemic-driven drug repurposing. We included literature addressing translational outcomes and pharmacoeconomic evaluations, with emphasis on studies from high-income country (HIC) settings where robust cost-effectiveness data were available.

A narrative review approach was chosen due to the diverse nature of the evidence base, which ranged from clinical trial reports to health economics analyses. This format allowed for conceptual synthesis across varied study designs and data types, facilitating a holistic integration of findings. The narrative approach was considered well-suited to distill broad translational lessons and pharmacoeconomic insights that might have been lost in a more rigid systematic review framework.

Conceptual framework

Translational science is often depicted as a stepwise T0-T4 continuum, moving from basic discovery to population‐level impact [5]. During COVID-19, this spectrum collapsed into a rapid-fire pipeline where months, not years, separated laboratory insight from bedside application. Basic virology (T0) and preclinical screening (T1) proceeded almost in parallel with early human studies (T2); pivotal trials (T3) and guideline adoption (T4) followed at record speed. For context, the median interval required to advance an infectious‑disease candidate from Phase II proof‑of‑concept to Phase III pivotal testing was ≈six to seven years before 2020, whereas the Randomised Evaluation of COVID‑19 Therapy (RECOVERY) and SOLIDARITY platforms delivered practice‑changing mortality data within roughly six to nine months of first patient enrolment. Three mechanisms drove this compression.

**Figure 2:**
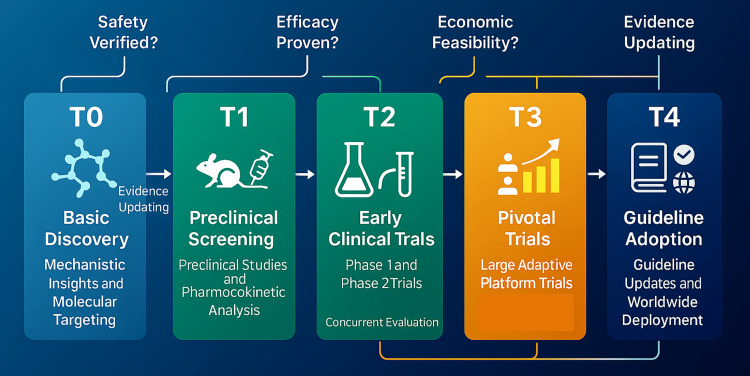
Adaptive translational pipeline for pandemic drug repurposing Color‑coded schematic of the compressed T0–T4 continuum: basic mechanistic discovery (T0), pre‑clinical screening (T1), early clinical studies (T2), pivotal adaptive trials (T3), and guideline adoption with worldwide deployment (T4); iterative evidence‑updating arrows illustrate feedback between stages.

First, adaptive platform trials such as RECOVERY in the United Kingdom [6] and SOLIDARITY under the World Health Organization [7] replaced stand-alone, single-drug studies. By testing multiple candidates concurrently and using response-adaptive randomization, these platforms generated decisive signals while conserving patients and resources. Ineffective agents were dropped swiftly, allowing promising drugs to graduate through the pipeline without restarting a new trial. The platform design thus became a translational escalator, continually feeding updated evidence to clinicians. Figure 2 visualizes the streamlined T0-T4 pathway that enabled rapid evaluation and global rollout of repurposed COVID‑19 therapies.

Second, the pandemic normalized the use of real-world evidence. Large electronic-health-record networks, hospital discharge datasets, and national registries supplied near-real-time safety and effectiveness data that complemented randomized evidence [8]. Although observational findings demanded careful adjustment for bias, they provided early reassurance or early warning, before full trial reports appeared. Living meta-analyses and guideline panels integrated these data in iterative updates, giving front-line physicians an evolving map rather than a static compass.

**Figure 3:**
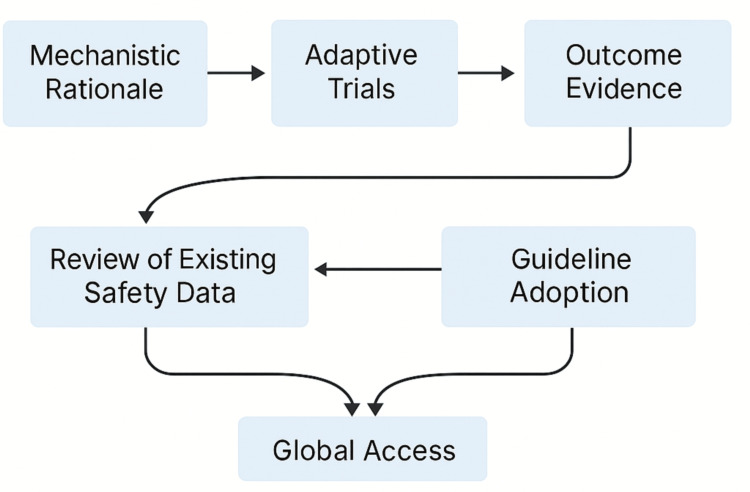
Evidence‑feedback workflow linking mechanistic rationale to global access Diagram illustrates the iterative cycle: mechanistic rationale progresses through adaptive trials and outcome evidence, which feed back to existing safety reviews and guideline adoption; convergent arrows indicate how these streams culminate in broad global access to repurposed therapies.

Third, the urgency of the crisis brought health-economic value lenses into the translational conversation far earlier than usual. Cost-utility modelling, budget-impact projections, and incremental cost-effectiveness ratios (ICERs) were generated contemporaneously with clinical outcomes [9]. Stakeholders sought to know not only whether a repurposed agent worked, but whether its benefit justified the inevitable trade-offs in strained hospital budgets. Dexamethasone exemplified a high-value win: profound mortality benefit paired with minimal acquisition cost [4]. By contrast, remdesivir yielded modest clinical gains at markedly higher prices, prompting nuanced purchasing decisions [10]. Embedding these economic appraisals within the translational pipeline ensured that resource allocation reflected both scientific merit and fiscal sustainability balancing act especially salient for high-income countries grappling with skyrocketing pandemic expenditures. Collectively, these innovations forged a conceptual framework where clinical efficacy, implementation feasibility, and economic value co-evolved, redefining what it means to translate a therapy from crisis conception to real-world cure. Figure 3 depicts the evidence‑feedback loop that operationalizes rapid translation from laboratory insight to worldwide deployment.

Mechanistic rationale & biological plausibility

Knowledge of SARS-CoV-2 pathophysiology provided a roadmap for pandemic-driven drug repurposing. The virus’s entry via the ACE2 receptor (facilitated by the host protease TMPRSS2) suggested that blocking these early steps might prevent infection [11]. This insight spurred trials of entry inhibitors (e.g., camostat mesylate) [12]. As infection progressed, immune-driven damage became prominent: uncontrolled inflammation (a “cytokine storm”) and endothelial injury with microthromboses emerged as hallmark features of severe COVID-19 [13,14]. Recognizing these processes, clinicians repurposed anti-inflammatory drugs (e.g., corticosteroids, IL-6 inhibitors) and anticoagulants (e.g., heparin) to mitigate the cytokine surge and coagulopathy [11]. By the same token, direct antiviral drugs were expected to be most beneficial in the infection’s early phase (before severe inflammation), consistent with findings that agents like remdesivir showed greater impact when used during initial viral replication.

Mechanistic rationale aligned with clinical success in several cases. The clearest example was the inexpensive corticosteroid dexamethasone, long used for its broad anti-inflammatory effects [3]. By dampening aberrant immune responses in patients with respiratory failure, dexamethasone significantly reduced 28-day mortality in hospitalized COVID-19 patients​ [6]. Likewise, blocking IL-6 with tocilizumab (an arthritis drug) improved survival among patients with escalating inflammatory markers when added to standard care ​[15]. These outcomes validate the strategy of targeting key inflammatory pathways implicated in COVID-19 pathology. Notably, both dexamethasone and tocilizumab had extensive pre-pandemic safety data, which helped clinicians deploy them rapidly once efficacy emerged.

Conversely, some biologically plausible therapies failed to demonstrate clinical benefit. Hydroxychloroquine, despite in vitro antiviral activity and theoretical immunomodulatory effects, did not improve outcomes in rigorous trials ​[16]. Similarly, multiple well-powered trials confirmed no meaningful benefit of ivermectin in COVID-19 patients [16]​. These cases underscore that even compelling mechanisms (or promising cell-culture data) do not guarantee efficacy, especially if effective drug concentrations are unattainable in vivo or if the intervention addresses the wrong phase of illness.

When prioritizing candidates for trials, pre-existing safety and pharmacokinetic profiles were critical. During the crisis, agents with established human safety records rose to the forefront, since their dosing and side effect profiles were already well characterized in humans. This enabled rapid trial launches under ethical oversight. Furthermore, pharmacokinetic modeling helped focus efforts: one analysis found only 14 of 56 proposed repurposed drugs were likely to reach effective antiviral levels in vivo, and those predicted to fall short were deprioritized ​[17].

Clinical outcomes evidence in pandemic drug repurposing

Table 1 synthesizes the pivotal trial outcomes that determined which repurposed drugs advanced to guideline endorsement and which were abandoned. Anti-inflammatory corticosteroids delivered the clearest survival benefit among repurposed COVID-19 therapies. In the large RECOVERY trial, dexamethasone (6 mg daily) significantly reduced 28-day mortality in hypoxemic patients: mortality was ~41.4% vs 29.3% in ventilated patients and 26.2% vs 23.3% in oxygen-only patients (rate ratios ~0.64 and 0.82)​ [6]. In the same RECOVERY trial, a low‑dose course of dexamethasone (6 mg daily) reduced 28‑day mortality by 36 % in ventilated patients and 18 % in those on supplemental oxygen, yet costs < US$ 1 per treatment-day unrivalled value proposition among all COVID‑19 therapies to date. No benefit was observed in patients not requiring respiratory support ​[17]. Meta-analyses corroborated a mortality reduction in severe COVID-19 (pooled OR ~0.65) ​[18], cementing low-dose steroids as the standard of care in severe disease.

**Table 1:** Summary of clinical outcomes of key repurposed drugs IL‑6: interleukin 6; JAK: Janus kinase; mAb:  monoclonal antibody; RNA: ribonucleic acid; RECOVERY: Randomised Evaluation of COVID‑19 Therapy; REMAP‑CAP: Randomized, Embedded, Multifactorial, Adaptive Platform Trial for Community‑Acquired Pneumonia; ACTT: Adaptive COVID‑19 Treatment Trial; RCT: randomised controlled trial; ↓: decrease; ↑: increase; WHO: World Health Organization.

Drug		Mechanism	Pivotal Trial	Primary Outcome	WHO Status
Dexamethasone [27]	Glucocorticoid (anti‑inflammatory)		RECOVERY 2020	↓ 28‑day mortality in ventilated patients	Recommended (severe/critical)
Tocilizumab [22]	IL‑6 receptor mAb		RECOVERY + REMAP‑CAP 2021	↓ death/ventilation composite	Recommended (with steroids)
Baricitinib [28]	JAK 1/2 inhibitor		ACTT‑2 2021	↑ recovery; ↓ progression	Recommended (hospitalized severe)
Remdesivir [27]	RNA polymerase inhibitor		ACTT‑1 2020	↓ time‑to‑recovery; no clear mortality ↓	Conditional (non‑severe, high‑risk)
Hydroxychloroquine [26]	Antimalarial		RECOVERY 2020	No mortality or clinical benefit	Strongly not recommended
Ivermectin [29]	Antiparasitic		Multi‑RCT 2020‑22	No consistent clinical benefit	Not recommended outside trials

Among antivirals, remdesivir showed only modest effects. In the NIH ACTT-1 trial, remdesivir significantly shortened time to recovery (median 10 vs 15 days), reflecting hospital length of stay, although the mortality reduction was not statistically significant [19]. However, the WHO SOLIDARITY trial found no mortality benefit​ [7] and only a small effect on hospital discharge ​[20]. Even pooled data confirmed only a minor impact: the composite of death or progression to ventilation occurred in 19.6% on remdesivir vs 22.5% on control (RR ~0.84), without a clear survival gain. Other repurposed antivirals also failed: lopinavir-ritonavir and interferon were halted for futility ​[20], and hydroxychloroquine showed no clinical benefit on mortality.

IL-6 receptor antagonists yielded mixed results. One randomised controlled trial (RCT) showed that tocilizumab cut the risk of mechanical ventilation or death by day 28 (12.0% vs 19.3%; HR ~0.56)​ [21], though overall 28-day mortality was not different. Subsequent pooled analyses suggest a modest survival benefit when tocilizumab is added to corticosteroids, especially in patients requiring oxygen or noninvasive support ​[22]. The benefit in already-intubated patients remains uncertain, emphasizing careful patient selection. Indeed, a Bayesian meta-reanalysis estimated >95% probability that tocilizumab reduces mortality in patients on oxygen only. These findings guide the use of IL-6 blockade in severe COVID-19.

Anticoagulation trials showed context-dependent effects. In moderately ill patients (non-ICU), therapeutic-dose heparin increased days alive without organ support and raised the probability of survival to discharge (absolute benefit ~4 percentage points) compared to standard prophylaxis​ [23]. By contrast, in critically ill patients, full-dose heparin conferred no improvement in survival or ventilator-free days over prophylaxis ​[24]. Major bleeding remained rare (<4%) but was higher with therapeutic dosing. Thus, most guidelines now reserve full anticoagulation for non-ICU COVID-19 patients.

Interest in selective serotonin reuptake inhibitors (SSRIs) stems from their putative anti-inflammatory effects. A meta-analysis of three trials (n≈2200) found that fluvoxamine (100 mg twice daily) reduced clinical deterioration or hospitalization by ~31% [relative risk (RR) ~0.69, 95% confidence interval (CI) 0.54-0.88] ​[25]. However, analyses focused on hospitalization alone showed only a non-significant 21% risk reduction. Observational data in large cohorts also noted lower mortality among patients on fluoxetine or fluvoxamine (RR ~0.72) ​[25]. These signals suggest SSRIs may prevent progression in early disease, but definitive RCT evidence on hard endpoints is still pending.

Finally, several repurposed agents were explicitly de-implemented after null results. Both RECOVERY and SOLIDARITY found no mortality or clinical benefit from hydroxychloroquine, lopinavir-ritonavir, or interferon, leading to early trial closures​ [20]. Convalescent plasma likewise failed to improve outcomes: RECOVERY reported identical 28-day mortality (24% vs 24%) and no faster discharge or reduced intubation ​[26]. These negative findings have led WHO and other authorities to advise against the routine use of these therapies. In contrast, drugs with proven benefit - e.g., dexamethasone (and tocilizumab in select patients)​​ [6,22] - are now embedded in treatment guidelines, illustrating how large trials have sharply defined which repurposed drugs succeed and which should be abandoned.

Pharmacoeconomic impact in high-income countries

In high-income countries (HICs), formal pharmacoeconomic frameworks were rapidly invoked to inform COVID-19 drug repurposing decisions. Decision-makers recognized the need to quantify the value of new or repurposed therapies even under emergency conditions ​[9]. For example, U.S. evaluators (ICER) developed dual pricing models for remdesivir - one “cost-recovery” approach based on manufacturing costs, and one based on traditional cost-effectiveness analyses​ [30]. These models explicitly contrasted covering production expenses with rewarding clinical benefit. In practice, agencies debated whether to apply usual willingness-to-pay (WTP) thresholds or adopt stricter criteria during the pandemic. ICER notably applied a lower $50,000/quality‑adjusted life‑year (QALY) cutoff for remdesivir, arguing that a tighter threshold was more “policy relevant” for broad public health use [31]. In essence, HIC decision makers acknowledged that, in a fast-moving crisis, conventional benchmarks might be adjusted to keep treatments affordable.

Pricing and perceived value differed greatly between low-cost generics and expensive novel therapies. Widely available, cheap drugs like dexamethasone had very favorable economics. One UK analysis found that rolling out dexamethasone nationwide would cost only about £940 per life-year gained - far below NICE’s ~£20,000/QALY threshold​ [32] - making it “clearly cost-effective.” By contrast, costly biologics or antivirals require much larger health gains to justify their price. Multiple economic reviews echoed this pattern: remdesivir’s per-course cost ranged widely (as little as $10 up to $780), but in most studies, its incremental cost-effectiveness fell below typical national thresholds (often only 8-23% of WTP) ​[33]. In sum, HIC analyses showed that inexpensive generics often delivered high value, whereas high-priced repurposed drugs underwent tighter value-based pricing scrutiny.

Analyses also highlighted differing priorities of hospitals, insurers, and societies. One U.S. cost-effectiveness model found that from a health-payer perspective (using bundled hospital payments), a hypothetical COVID therapy yielded about $22,933 per QALY, but including societal gains (productivity) nearly halved this to $11,492/QALY ​[9]. Under a fee-for-service hospital model, a $2,500 drug that shortened hospital stays by ~2.7 days produced roughly $19,469/QALY (dropping to $8,028 when societal impacts were counted)​. These numbers underline how perspective drives value judgments: hospitals emphasized immediate cost offsets (bed-days saved), while payers and society looked at lifetime benefits and broader economic impacts. In practice, governments and insurers in HICs ran real-time budget-impact models to gauge how many patients could be treated before exceeding spending caps. Notably, ICER’s framework considered the population budget impact in pandemic times, stressing that even a cost-effective drug could overwhelm the health budget if used widely ​[31]. In the U.S., where no single health budget or fixed WTP exists, policymakers grappled with this uncertainty, whereas in single-payer systems like the NHS, authorities could set explicit spending ceilings and allocate accordingly.

These tensions - getting drugs to patients quickly versus protecting limited budgets, and fair access versus cost containment - ran throughout the crisis. Emergency authorizations and stockpiling in early 2020 (e.g., U.S. federal contracts) often bypassed standard economic review to maximize access. Yet as data emerged, HTA bodies re-engaged, using cost-effectiveness and budget models to renegotiate prices or restrict use. As ICER observed, “treatments must be affordable to meet the shared goal of defeating COVID-19 [34]. In several cases, value-based pricing emerged: drugs were priced at the top of what health systems could afford, given their benefit. For example, in the U.S. framework analysis, the value-based price of a hypothetical treatment with the modelled profile was estimated at $37,710 under a $100,000/QALY ceiling​ [9]. Ultimately, economic tools like ICER’s threshold analyses and budget-impact projections guided resource allocation. They helped shape policies that, for instance, earmarked high-cost therapies for patients most likely to benefit while deploying cheap generics widely. In HICs, these pharmacoeconomic lessons underscored that even amid an emergency, drug repurposing decisions had to balance speed and equity with fiscal prudence.

Implementation & uptake

In high-income countries, guidelines (eg, NIH, WHO) evolved rapidly as evidence on repurposed COVID-19 therapies accumulated. Early recommendations drew on retrospective cohorts and small trials, but pragmatic adaptive platform trials (eg, RECOVERY) later provided more definitive data, prompting updates to guidance [35]. This rapid evidence-to-guidance cycle varied by region, but ultimately helped ensure proven therapies were recognized once trial results emerged. In practice, agencies issued frequent interim updates (for example, WHO published seven versions of its COVID-19 therapy guideline in 2020) to reflect new data.

Real-world adoption was uneven. Many clinicians initially prescribed off-label agents (eg, hydroxychloroquine) amid early enthusiasm [36], whereas others awaited formal endorsements. Hospitals with active stewardship programs often imposed formulary restrictions that aligned practice with updated guidance [37]. Indeed, surveys found remdesivir was widely used but often deviated from NIH recommendations, except where stewardship oversight was in place. Global shortages of some drugs (eg, antimalarials) also influenced timing and access.

Stewardship frameworks and digital tools were critical for oversight. Many health systems reconfigured antimicrobial stewardship modules or EHR dashboards to track COVID-19 therapies in real time [38]. Challenges remained. The fractured, fast-changing information environment often confuses both clinicians and patients [39]. Politicized debates and media hype around certain repurposed drugs (eg, hydroxychloroquine, ivermectin) polarized attitudes [36]. Some patients hesitated to try novel therapies without stronger assurances, while others pressured clinicians for unproven treatments. In sum, uptake in HICs was mediated by evolving evidence, stewardship efforts, and the broader information landscape.

Knowledge gaps & lessons

The pandemic exposed persistent knowledge gaps. Many early repurposing trials relied on surrogate endpoints (such as viral clearance or biomarkers) rather than hard clinical outcomes, complicating interpretation. Real-time data sharing was limited: one analysis found only about sixteen percent of COVID-19 trials planned to share their raw data [40]. An “infodemic” of misinformation further clouded judgment: disproven treatments (e.g., hydroxychloroquine) continued to be championed on social media and by some officials even after rigorous trials showed no benefit [41]. However, some strategies proved effective. Large adaptive platform trials (such as the RECOVERY and SOLIDARITY trials) rapidly tested multiple repurposed drugs in parallel, delivering clear evidence on what did and did not work [42]. These well-powered RCTs, using mortality or recovery as endpoints, cut through noise. In contrast, observational signals often proved unreliable: early excitement about therapies like convalescent plasma faded when later trials showed no survival benefit. This reinforced the need to default to “core principles of evidence-based medicine,” even in crises [42].

Translational science lessons suggest better preparedness. Future pipelines should integrate robust in vitro and animal testing with pharmacokinetic and pharmacodynamic modeling to prioritize candidates effectively [43]. Embedding health-economic analysis early in trial design would ensure only cost-effective therapies advance. Finally, proactive science communication and policies to counter misinformation are vital, since trust in evidence-based guidance is as crucial as the science itself.

Future directions & recommendations

To prepare for future pandemics, coordinated policy and infrastructure reforms are needed. For example, experts recommend building a maintained library of off-patent drugs with known safety records and proactively cataloging their potential antiviral uses for rapid screening [44]. Policymakers might establish pre-negotiated licensing agreements or global stockpile systems to prevent richer countries from monopolizing effective treatments. Embedding health economics into trial design is also important to ensure candidate therapies are cost-effective and accessible.

Translational and economic reforms should go hand in hand. Governments and funders could increase investment in broad-spectrum antivirals and use advanced purchase commitments to incentivize the production of promising repurposed drugs. Regulatory agencies should harmonize emergency-use authorizations and agree on simple master trial protocols to launch studies immediately. Incorporating cost-effectiveness analysis early would ensure that only financially sustainable options are pursued. In parallel, boosting manufacturing capacity is also critical: WHO noted that Africa’s poor local production and import reliance worsened pandemic shortages [45].

Finally, global coordination must be strengthened. Proposals include an international data-sharing hub for outbreak therapeutics, pooling trial results and epidemiologic data in real time. A global repurposing consortium could align research priorities, share compound libraries, and coordinate multicenter trials.

## Conclusions

Lessons from COVID‑19 demonstrate that repurposing can move swiftly from bench to bedside when guided by rigorous evidence, collaboration, and equity. Cheap generic therapies-such as dexamethasone-proved lifesaving when validated by well‑run trials, whereas hype‑driven treatments squandered resources. The pandemic highlighted the importance of large platform studies, open data, and countering misinformation. Economically, it underscored that even off‑patent drugs require proactive policies to secure global access. By embedding health‑economic evaluation and strengthening international agreements, crisis‑driven innovation today can be converted into sustained preparedness for tomorrow.
